# Human Schistosomiasis Vaccines as Next Generation Control Tools

**DOI:** 10.3390/tropicalmed8030170

**Published:** 2023-03-14

**Authors:** Peter J. Hotez, Maria Elena Bottazzi

**Affiliations:** 1 Texas Children’s Hospital Center for Vaccine Development, Departments of Pediatrics and Molecular Virology, National School of Tropical Medicine, Baylor College of Medicine, Houston, TX 77030, USA; 2 Department of Biology, Baylor University, Waco, TX 76798, USA; 3 James A Baker III Institute of Public Policy, Rice University, Houston, TX 77005, USA; 4 Hagler Institute for Advanced Study and Scowcroft Institute of International Affairs, Texas A&M University, College Station, TX 77840, USA

## 1. Introduction

Human schistosomiasis remains one of the most important yet neglected tropical diseases, with the latest estimates from the Global Burden of Disease Study indicating that over 140 million people are infected with schistosomes [1], although other estimates are considerably higher [2]. More than 90% of the schistosomiasis disease burden occurs on the African continent, where this infection disproportionately affects female reproductive health [3], while, in both Africa and Brazil, schistosomiasis causes chronic hepatic or intestinal illnesses and malnutrition [4].

For almost 20 years, the major approach to schistosomiasis has relied on the regular and periodic mass treatment of school-aged children with an annual dose of praziquantel. A major policy breakthrough was an agreement by the German pharma company, Merck KgaA, to donate praziquantel for this purpose, such that 250 million tablets of praziquantel are now donated annually [5]. The World Health Organization (WHO) leads coordination efforts for the mass drug administration of schistosomiasis in a network of treatment programs for at least 10 neglected tropical diseases [6,7]. Mass drug administration for both major forms of schistosomiasis caused by Schistosoma haematobium (urogenital disease) and Schistosoma mansoni (intestinal-hepatic disease) is tailored or modified depending on the local or regional prevalence of these illnesses. For example, in areas where the schistosomiasis prevalence among school-aged children exceeds 50%, annual treatment is recommended, whereas mass drug administration can be carried out every other year or even every three years where the prevalence is lower [8]. Moreover, in high prevalence areas, the WHO recommends treating adults [8].

In 2021, the WHO estimated that more than 75 million received Merck KgaA-donated praziquantel through mass treatment programs, also referred to as preventive chemotherapy [9]. This includes almost 60 million school-aged children and 16 million adults, numbers that comprise roughly 30% of those requiring treatments [9]. A major question is whether mass treatment alone will suffice in reducing the global disease burden of schistosomiasis or whether additional or companion technologies will be required.

## 2. Opportunities for “Vaccine-Linked Chemotherapy”

At the end of the 2022, the International Task Force for Disease Eradication (ITFDE) met at the Carter Center (Atlanta, GA, USA) to conclude that praziquantel mass drug administration as a single intervention may not be adequate for eliminating schistosomiasis in Africa or globally [10]. Such conclusions are based on operational research projects demonstrating the persistence of infection and transmission despite multi-year mass treatments with praziquantel [10], together with epidemiologic modeling studies showing the need to treat both children and adults [11,12]. Still another concern is whether the generous 250 million tablet donation from Merck KgaA is even adequate given the scope and magnitude of the schistosomiasis disease burden in both adults and children [10], or whether additional drugs such as oxamniquine or metrifonate may be required to enhance drug effectiveness [13].

Further modeling from Dr. Klodeta Kura, working with Professor Roy Anderson and colleagues at Imperial College, London, found adding schistosomiasis vaccines to praziquantel mass treatment programs to be beneficial. Specifically, an effective vaccine in concert with mass treatment could reduce the likelihood of reinfection or hasten the elimination of parasite transmission [14,15]. Their studies also confirmed the cost-effectiveness of schistosomiasis vaccines. Thus, programs of “vaccine-linked chemotherapy”, first proposed in 2005 by Drs. Robert Bergquist, Lydia Leonardo, Graham Mitchell, and others, could offer opportunities to reduce the disease burden of schistosomiasis and facilitate its elimination as a public health problem [16]. Thus, human schistosomiasis vaccines could be integrated within the organized Global Schistosomiasis Alliance (https://www.eliminateschisto.org/ (accessed on 24 February 2023)) strategy, comprised of preventive chemotherapy with praziquantel, together with programs of operational research, to maximize the use of current and new control tools.

## 3. The Current Pipeline

Because *S. haematobium* and *S. mansoni* co-infections are widespread across Africa [17], an effective vaccine would likely need to target both major schistosome species. However, schistosome infections caused exclusively by *S. mansoni* remain a significant public health threat in Brazil, and possibly in Suriname and Venezuela [18]. In Asia, *Schistosoma japonicum* in infections account for less than 1% of the global disease burden.

There are currently three schistosomiasis vaccines currently undergoing product development and clinical testing. Each one was developed to target *S. mansoni*, with an intent or desired product profile to have them cross-protect against S. haematobium. In addition, a fourth candidate vaccine that was specifically developed to target S. haematobium upon completed clinical testing but was ultimately abandoned due to lack of efficacy (despite its immunogenicity) in phase 3 clinical trials conducted in Senegal [19].

Sm-TSP-2. The Texas Children’s Hospital Center for Vaccine Development, a product development partnership for neglected tropical disease vaccines, together with academic partners at George Washington University, James Cook University, and the Seattle-based Access to Advanced Health Institute (AAHI), has developed a recombinant protein vaccine that targets a surface tetraspanin of *S. mansoni* [20,21,22,23,24]. The antigen was discovered through immunomics profiling and was shown to be protective in a laboratory mouse challenge model, as evidenced by reductions in liver or fecal parasite egg counts and worm pairs [20], before it was scaled up for production in yeast [21,22] and formulated on Alhydrogel together with synthetic lipid A (glucopyranosyl lipid A, GLA). The vaccine was found to maintain its potency during animal testing [23] and was immunogenic in phase 1 trials [24]. It has since advanced to phase 2 clinical testing in Uganda.

Sm14. The Oswaldo Cruz Institute of FIOCRUZ (Oswaldo Cruz Foundation), together with the Ludwig Institute for Cancer Research, Cornell University, and AAHI, has developed a second vaccine that targets *S. mansoni*. It is comprised of a recombinant protein 14 kDa fatty acid-binding protein, which is also formulated with GLA but in a stable emulsion and without an aluminum-containing adjuvant [25]. This vaccine has also completed phase 1 clinical testing, and was also shown to cross protect against fascioliasis, an important veterinary parasitic infection [25,26,27].

Sm-p80. PAI Life Sciences, a Seattle-based biotech, in collaboration with Texas Tech University, AAHI, and the International Vaccine Institute in Korea, has developed a third recombinant protein vaccine candidate known as Sm-p80 [28,29,30,31,32,33]. This molecule corresponds to a *S. mansoni* calcium-activated protease (calpain) also found in schistosome tegument or surface. It was selected because of its protective efficacy in a baboon challenge model—as evidenced by reductions in parasite egg load and hatching as well as adult worm pair antigen—together with its cross protection versus *S. haematobium* [28,29,30,31,32,33]. Similar to the Sm14 vaccine, Sm-p80 is expressed in Escherichia coli bacteria and formulated in a stable emulsion with GLA. This vaccine has recently begun clinical testing.

Currently, these vaccines are being tested separately in clinical trials. However, given their potentially similar modes of action—two of the three candidates are schistosome surface proteins—and the fact that each employs the same GLA adjuvant, there is a rationale for evaluating them together. For example, there may be additional protective immunity gained by combining two or more antigens in a bivalent or a trivalent vaccine. There is also potential cost-savings and economy of scale by testing all three antigens at the same time and in clinical trials run by the same clinical trial team. Another opportunity would be to test these antigens simultaneously using alternative adjuvants. For instance, the Novavax Matrix M adjuvant that is being used for a new malaria vaccine produced by the Serum Institute of India [34] might also be useful for any of the three schistosome antigens. As discussed below, doing so might afford opportunities for co-formulating malaria and schistosomiasis vaccine antigens for Africa where both tropical infections are co-endemic.

Another aspect of human schistosomiasis vaccine development is how improvements in bioinformatics, immunomics, and computational biology might be able to identify next-generation recombinant antigens based on completed schistosome genomes [35,36]. Moreover, cutting-edge biological techniques such as single-cell sequencing [37] could revolutionize our understanding of schistosome developmental biology and therefore identify new and exciting vaccine targets [38]. A key point here is that our very best schistosome vaccine candidates may remain undiscovered. Another concern is the emergence of hybrid worm pairings in Africa between *S. haematobium* and *S. mansoni*, or *S. haematobium* and *S. intercalatum* or other zoonotic schistosome species [39]. The impact of such biological hybridization on the evolution of potential vaccine antigens requires additional studies.

To date, all three schistosomiasis vaccines are based on recombinant proteins. However, there is the potential to explore these antigens with alternative vaccine delivery technologies, including mRNA vaccines. A paper proposing schistosomiasis mRNA vaccines was one of the last written by the late Professor Donald McManus before his passing at the end of 2022 [40]. Prof. McManus also pursued both human and veterinary vaccines for Asian schistosomiasis, including comparisons of some of the orthologous antigens in development for S. mansoni and S. haematobium [41]. An alternative would be to evaluate prime–boost approaches that mix technologies including mRNA with protein-based vaccines or other technologies [42]. This could also include adenovirus–virus vectored approaches or creating immunogenic nanoparticles. The portfolio of new and innovative technologies that has benefited the development of COVID-19 pandemic vaccines has yet to translate into changes in portfolios or portfolio management for new and neglected tropical disease vaccines.

## 4. Future Directions

**Figure 1:**
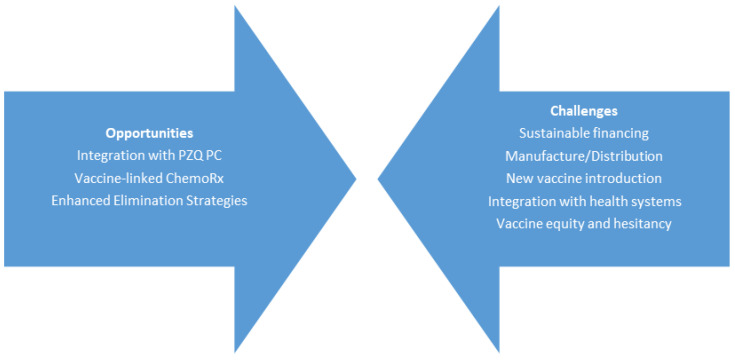
The opportunities versus challenges of human schistosomiasis vaccines. Abbreviations: PZQ (praziquantel), PC (preventive chemotherapy), ChemoRx (chemotherapy).

Even as these three schistosomiasis vaccines advance through scale-up process development and clinical evaluation, the challenges to ensure that they are produced at an industrial scale, tested in pivotal phase 3 trials, and ultimately integrated into an appropriate health system, are formidable (Figure 1).

Among them will be the sustainable financing required and the cooperation of a multinational pharma company or vaccine producer from the Developing Country Vaccine Manufacturers Network (https://dcvmn.org/ (accessed on 24 February 2023)) based in low- and middle-income countries [43]. To date, no major vaccine producer has shown strong interest in the industrial-scale production of a human schistosomiasis vaccine, or really any anthelminthic vaccine. One reason for this situation is the absence of a guaranteed purchaser of the vaccine or any of the advanced market commitments that were successful for COVID-19 and other immunizations. Even though (based on modeling studies) a human schistosomiasis would be cost-effective [44], this feature alone cannot ensure adequate investments from the private sector, nor financial support either from national governments or global health policymaking bodies. These realities apply for many of the other neglected tropical disease vaccines in development; an exception has been a human dengue vaccine that might find use in both high-income and low-income countries [43].

A new Coalition for Epidemic Preparedness Innovations (CEPI) (https://cepi.net/ (accessed on 24 February 2023) could lead sustainable financing efforts, although, to date, this organization has focused its attention primarily on countermeasures for pandemic threats rather than neglected tropical diseases. Making a case with the CEPI for supporting vaccines to prevent chronic, neglected, and debilitating parasitic infections, such as schistosomiasis, could become an important step in sustainable financing. Overall, there is an urgent need to bring together the global policymakers, donor community, and a coalition of vaccine producers to address the problem of vaccine equity as it applies to neglected tropical disease burdens. This approach has been somewhat successful for vaccines to combat pandemic threats, but not yet the chronic and debilitating conditions.

An equally formidable strategy involves identifying an appropriate health system to deliver a schistosomiasis vaccine alongside other pediatric vaccines for global health. Because of its impact on women’s reproductive health, any schistosomiasis vaccine would need to sustain protection into adolescence and early adulthood. This may require periodic boosting, potentially alongside praziquantel mass drug administration. On that basis, co-administering a human schistosomiasis vaccine with the human papillomavirus (HPV) vaccine represents an attractive means to improve the health of girls who live in extreme poverty. Together, schistosomiasis, human papillomavirus (HPV) infection, and human immunodeficiency virus/acquired immune deficiency syndrome (HIV/AIDS) comprise three of the most important chronic and debilitating conditions of these populations [45]. Therefore, schistosomiasis control could comprise a triumvirate of innovations specifically directed to the plight of girls and women on the African continent.

Another possibility would be to administer a schistosomiasis vaccine together with a malaria vaccine, given the high co-endemicity of schistosomiasis and malaria on the African continent [46]. Two malaria vaccines are now being introduced in Africa [34]. Another option is to co-administer schistosomiasis and human hookworm vaccines, recognizing that, together, malaria, schistosomiasis, and hookworm comprise the leading causes of anemia in resource-poor settings [47]. Therefore, targeting anemia through vaccinations could represent an attractive option for policymakers. This is especially true for pregnant women who suffer from profound and significant anemia in areas where these infections are syndemic [48].

Finally, it is worth noting that rising vaccine hesitancy in North America and Europe is extending into low- and middle-income countries, including those where schistosomiasis is endemic [49]. Combating vaccine hesitancy and refusal around malaria vaccine introduction has become a new challenge [34], and we should anticipate this might occur for schistosomiasis vaccines.

Unlike the reductions in Asian schistosomiasis in Japan and in some areas of China, which are due to accelerated rural economic development, it is less likely that similar economic drivers will reduce the prevalence and intensity of schistosomiasis on the African continent or in the most impoverished areas of the Americas anytime soon [50]. Therefore, in addition to continuing mass drug administration programs with praziquantel, introducing new schistosomiasis vaccines will become an essential component for disease elimination.

## Data Availability

No new data was created for this opinion article.
